# Access to root canal treatment in a Nigerian sub-population: assessment of the effect of dental health insurance

**DOI:** 10.4314/ahs.v21i1.57

**Published:** 2021-03

**Authors:** Paul Ikhodaro Idon, Olawale Akeem Sotunde, Temiloluwa Olawale Ogundare, Janada Yusuf, John Oluwatosin Makanjuola, Abdulmumini Mohammed, Chibuzor Emmanuel Igweagu, Olusegun Alalade

**Affiliations:** 1 Department of Dental Surgery, University of Maiduguri Teaching Hospital, Maiduguri, Nigeria; 2 Department of Restorative Dentistry, Faculty of Dentistry, Bayero University, Kano, Nigeria; 3 Department of Restorative Dentistry, Obafemi Awolowo University Teaching Hospital Complex, Ile Ife, Nigeria; 4 Department of Restorative Dentistry, College of Medicine, University of Lagos, Idi Araba, Surulere, Lagos State, Nigeria

**Keywords:** Dental insurance, health insurance, root canal treatment

## Abstract

**Background:**

The final pathway of tooth mortality lies between tooth extraction, and the more expensive and less accessible root canal treatment (RCT).

**Aim:**

To determine the extent to which individuals' financial resources as measured by socioeconomic status and dental insurance coverage affects their access to RCT.

**Methods:**

A hospital-based study that used a 15-item questionnaire to collect data among patients scheduled for RCT. All scheduled subjects (N = 291) over a one-year period constituted the sample for the study. Using the SPSS software, associations between the subjects' variables, and the dental insurance status were carried out with Chi square and independent t test respectively at 95% confidence interval.

**Results:**

Two hundred and ninety-one subjects were to have 353 RCTs within the study period. A high proportion (79.7%, p < 0.001) of the subjects had dental health insurance, majority (95.3%) of which was government funded. 20.9% of those with previous tooth loss was due to inability to afford cost of RCT. The lowest socioeconomic group had the highest proportion (90%, p = 0.421) of insured that visited for RCT.

**Conclusion:**

Dental insurance increased access to RCT. Socioeconomic status did not affect dental insurance status and dental visit for RCT.

## Introduction

Access to oral health care refers to an individual's ability to obtain and benefit from dental services provided by professionals in the dental care system, as the best way to achieve good oral health.^1^,^2^ Disparities however occur in access to oral health services in most populations. It is further aggravated by an indifferent public perception toward the necessity of dental services, fear of dental professionals and dental procedures, unequal distribution of dental manpower and an inadequacy or absence of health insurance coverage for dental services.^3^–^8^ Where there is demand for these services, especially when prompted by symptoms, access then is usually determined by availability and affordability of the services. Where the dental health facilities are available to render service, the ability to finance treatment may pose a significant barrier to access.

Studies show that out-of-pocket costs remain the single most significant obstacle barring individuals from receiving oral health services, and this has been shown to be highly dependent on income.^3^,^9^,^10^,^11^ However, a major objective of a good health care system should be to provide every individual with access to care irrespective of income or ability to pay, but rather on the basis of need. This objective is not fully realized for dental services in most populations. Thus, the patient usually has the option of direct out-of-pocket payment or by insurance. Dental insurance removes the financial burden of dental treatment from the individual. This becomes an important motivator for behavior change that favors increased dental attendance and utilization of dental services among individuals who would have acted otherwise. 11 Wall and Brown (1999) in their review of the effect of dental insurance showed a significant increase in dental visits among Americans that was associated with the growth in employer-based private dental insurance. 12 Similarly, Miller and Locker (1999) not only reported that dental visits are determined by income and dental insurance but also that insured patients are more likely to receive dental care.^9^ Although most people may be able to access basic dental care without dental insurance, comprehensive specialized dental care which are more expensive are mostly out of reach of the uninsured and is therefore a barrier.^13^

In contrast to most developed countries, dental services in Nigeria are generally provided by dental clinics in public hospitals which are highly subsidized. Even so, the charges are still relatively expensive for the general population of dental patients who may not be able to afford it. Considering the contribution of oral health to overall health and quality of life, this financial barrier to accessing dental care poses significant threats to equal opportunities for access to comprehensive health care. The findings from Western populations show that access to dental care is influenced majorly by income and availability of dental insurance.^3^,^9^,^10^,^14^,^15^ To our knowledge, there is yet no Nigerian study to determine access to dental care as it relates to income or dental insurance coverage. However, there have been several isolated studies on utilization of dental services, reasons for visits and associated factors among specific sub-populations. 4,16 Some of these studies reveal that there is poor use of dental services, and most dental visits are due to pain from pulpal sequelae of untreated caries.^8^,^17^,^18^

Although the prevalence of dental caries is relatively low in Nigeria as compared to figures from developed countries, it still presents a significant burden as most go untreated.^4^,^16^ Presentation is therefore usually late with onset of pulpal and periapical sequalae. With the increasing prevalence of caries in the developing countries the need for treatment of these sequela is high as well.^19^ The final options for treatment in the presence of these sequelae, due to delays in presentation, are tooth extractions or endodontic procedures such as root canal treatment (RCT) which preserves the tooth in the mouth. RCT is a specialized endodontic dental treatment that requires expert management, and on the average is relatively expensive in most settings. In addition, root-treated teeth may require post endodontic restorations that are expensive, and may not be covered by dental insurance. In the context of financial barriers to receiving specialist dental services, most patients may rather go for tooth extraction, which is a cheaper option. This is further buttressed by the report of Barethin (1976) that insured patients have higher odds of receiving endodontic treatment than the uninsured.^20^ Thus, the lack of resources to afford endodontic treatment, as the final option to save the tooth, either as out-of-pocket payment or insurance may prevent access to this treatment option.

Health insurance in Nigeria is provided for the population, but majorly for the formal sector by the National Health Insurance Scheme (NHIS), and to a lesser extent by employer-based private insurance. The aim of establishing the NHIS was to give all Nigerians equal opportunity to access good quality health care. However, the scheme has so far only been able to account for about 3.5% of health expenditure, while out-of-pocket payment still stands at 90% of payments for health charges. 21 The range of oral health care services covered by the NHIS have increased over the years to include some preventive dental treatments, dental restorations, limited removable dental prosthesis, endodontic procedures and minor oral surgical procedures, with the exception of aesthetic treatments and fixed dental prosthesis.^22^ Unfortunately, there is no agreement on the exact list of dental procedures that are covered on the scheme. Thus, there is a discrepancy in the coverage allowed across the different hospitals in the country. Using the access to RCT, the only alternative to extraction in the final pathway of tooth mortality, this study aimed to determine the extent to which individuals' financial (socioeconomic status) resources and dental insurance coverage affect their access to this dental treatment.

## Methods

This study was carried out using a descriptive cross-sectional design following approval from the Research and Ethics Committee of the University of Maiduguri Teaching Hospital (UMTH) and Aminu Kano Teaching Hospital (AKTH), where data were collected. The study was conducted in full accordance with ethical principles including the World Medical Association Declaration of Helsinki (version 2008). Both centers are public tertiary health centers located in the Northeast and Northwest of Nigeria respectively, and have dental clinics with endodontic specialists and serve as referral centers for endodontic and restorative procedures in the regions. A 15-item questionnaire was designed, made available in printed form and administered to consenting subjects who were scheduled for RCT and met the inclusion criteria in the restorative clinics of both centers where RCTs are routinely carried out.

The questionnaire was administered to consecutive adult subjects attending the restorative clinic of UMTH (n = 166), and AKTH (n = 125) for RCT over a oneyear period. The first part of the 15-item questionnaire contained questions relating to participant's socio-demographics, including age, gender, marital status, occupation and possession of dental insurance. The occupation was classified into five classes, a modification of occupational strata devised by Famuyiwa et al. (1998)^23^: Class I (Executive managers, company directors, doctors, engineers, lawyers, university professors, traditional chiefs); Class II (Civil servants, nurses, teachers, secretaries, clergymen, businessmen, pensioners); Class III (Tailors, bricklayers, carpenters, typists, clerks, housewives); Class IV: (Messengers, roadside traders, cleaners, night guards, farmers, unemployed); and Class V (undergraduate students, postgraduate students). The second part of the questionnaire was used to collect information on number of teeth missing due to caries, and why RCT was not performed to save those teeth, number of teeth that requires RCT, and reasons for opting for RCT over extraction.

### Data management and analysis

Data obtained were coded and entered into the SPSS software version 23 (IBM SPSS Inc. Chicago, IL, USA). Descriptive statistics was generated to determine the proportions of the subjects' sociodemographic variables. The association between the subjects' variables and possession of dental insurance was done with Chi square test at 95% confidence interval. The independent t test was used to compare the mean number of RCTs by subjects' dental insurance status. The level of statistical significance was set at p< 0.05.

## Results

The participants of the study comprised of 291 adults whose ages ranged between 18 to 74 years, and included 127 females (43.6%) and 164 males (56.4%). For economic and social reasons, the subjects were categorized into three age groups, 18 – 34, 35 – 54, and 55 – 74. A larger proportion (69.4%) were younger than 35 years. Slightly more than half (54.3%) of the subjects were married. Only 3.4% of the subjects belonged to the Class IV occupational category, the lowest socioeconomic class (Table 1).

**Table 1:** Socio-demographic characteristics of subjects (N = 291)

Variable	n (%)
**Location**	
UMTH	166 (57.0)
AKTH	125 (43.0)
**Age (years)**	
18 – 34	202 (69.4)
35 – 54	77 (26.5)
55 – 75	12 (4.1)
**Gender**	
Male	164 (56.4)
Female	127 (43.6)
**Marital status**	
Married	158 (54.3)
Single	129 (44.3)
Widowed	3 (1.0)
Divorced	1 (0.3)
**Occupation**	
Class I	23 (7.9)
Class II	118 (40.5)
Class III	28 (9.6)
Class IV	10 (3.4)
Class V	112 (38.5)

A statistically significant (p < 0.001) proportion (232, 79.7%) of the subjects had dental health insurance that covered RCT. A significant majority (p < 0.001, 95.3%) had the government funded insurance. Among the insured subjects, about 87% claimed they would still have opted for RCT instead of extraction if they did not have dental insurance (Table 2). All the other subjects (30, 12.9%) that would have opted out of the treatment in the absence of dental insurance gave the high cost of RCT as their reason.

**Table 2:** Dental insurance data and preference for root canal treatment

Variable	N (%)	χ^2^	p
**Dental insurance**			
Yes	232 (79.7)	291.000	< 0.001
No	59 (20.3)		
**If yes, type of insurance**			
Government (NHIS)	221 (95.3)	311.846	< 0.001
Employer-based (private)	11 (4.7)		
**Would you choose RCT if you had no** **dental insurance?**			
Yes	202 (87.1)	281.439	< 0.001
No	30 (12.9)		

χ^2^ = Chi square

The highest proportion of insured subjects (65, 84.4%) by age was among the 35 – 64 age group, with the least (7, 58.3%) found in the 55 – 74 age group. The proportion of males (132, 80.5%) with insurance was similar to the females (100, 78.7%). About 65% of the subjects in the Class I occupational category had dental insurance, with the highest (90%) among the Class IV occupational category. None of the differences in proportion of the insured versus uninsured by the subjects' variables was found to be statistically significant, p > 0.05 (Table 3).

**Table 3:** Dental insurance data by subjects' variables

Variable	Dental insurance			χ^2^	p
			
	Yes = 232 n (%)	No = 59 n (%)	Total N = 291		
**Location**					
UMTH	135 (81.3)	31 (18.7)	166	0.612	0.434
AKTH	97 (77.6)	28 (22.4)	125		
**Age (years)**					
18 – 34	160 (79.2)	42 (20.8)	202		
35 – 54	65 (84.4)	12 (15.6)	77	4.479	0.107
55 – 74	7 (58.3)	5 (41.7)	12		
**Gender**					
Male	132 (80.5)	32 (19.5)	164	0.135	0.713
Female	100 (78.7)	27 (21.3)	127		
**Marital status**					
Married	130 (82.3)	28 (17.7)	158		
Single	99 (76.7)	30 (23.3)	129	6.042	0.110
Widowed	3 (100)	0 (0)	3		
Divorced	0 (0)	1 (100)	1		
**Occupation**					
Class I	15 (65.2)	8 (34.8)	23		
Class II	96 (81.4)	22 (18.6)	118		
Class III	22 (78.6)	6 (21.4)	28	3.893	0.421
Class IV	9 (90)	1 (10)	10		
Class V	90 (80.4)	22 (19.6)	112		

χ^2^ = Chi square

A total of 353 RCTs were to be carried out on the 291 subjects. The number of teeth for RCT per subject ranged from 1 to 6 with a mean of 1.21±0.51. The majority of these (97.9%) were to be carried out by conventional orthograde RCT. The mean RCTs to be performed was higher among subjects without dental insurance but statistically insignificant (p = 0.358) (Table 4).

**Table 4:** Mean number of root canal treatments

Dental insurance	n (%)	Mean number of RCTs	p
Yes	278 (78.8)	1.20±0.50	0.358
No	75 (21.2)	1.27±0.55	

n = number of teeth to be treated with RCT

**Figure 1:**
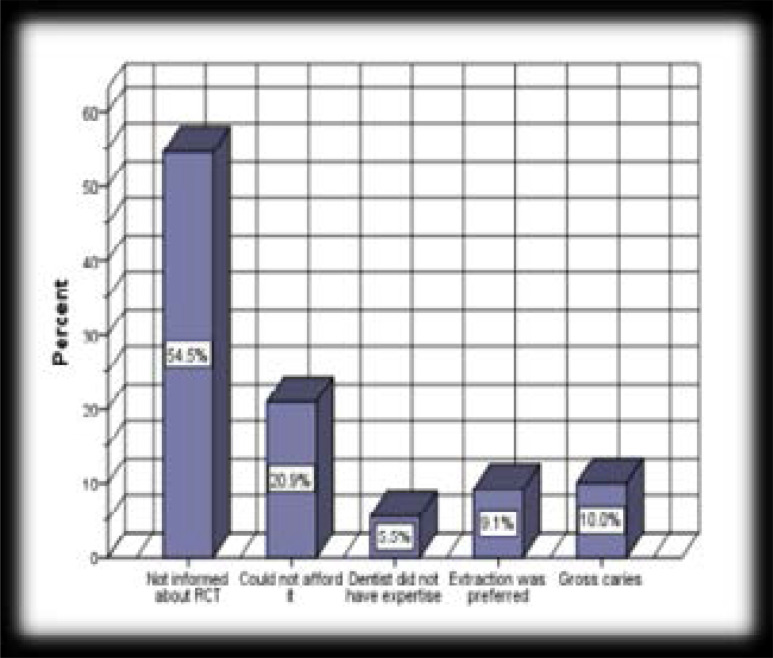
Reasons why RCT was not done to save missing teeth

The number of teeth missing due to caries ranged from 1 to 6 per subjects in 37.8% of the subjects. Figure 1 shows the reasons why RCT was not carried out to save these teeth. Not being able to afford the cost of RCT (20.9%) was only superseded by lack of information about RCT (54.6%) as an alternative to tooth extraction.

## Discussion

Endodontic treatment, particularly RCT is one of the fastest growing areas of dental practice. Idon and Yusuf (2018) in a retrospective study reported a considerable increase in the number of RCTs performed in one of the settings for the present study.^24^ One of the reasons suggested for this was the introduction of coverage of this procedure by the NHIS. The importance of the procedure lies in the avoidance of the alternative to it, which is tooth extraction. It is therefore likely that patients who could not afford it as out-of-pocket payment may opt for extraction. It would be safe to say that in the absence of cost as a barrier, and possession of insurance provided by the NHIS, individuals have access to more preferred range of dental treatments. This present study showed that 12.9% of those with health insurance would have declined RCT if they were not covered for it due to cost. Several authors have reported that tooth extractions were more frequently done by uninsured subjects.^35^,^25^–^27^ Brennan and Ellershaw (2012) also provided additional evidence when they reported that insured subjects and those on high income demanded more for routine dental treatment and comprehensive care.^28^ This present study also revealed that about one-fifth of the subjects with teeth missing due to caries did not go for RCT to save the lost teeth because of the cost, although their health insurance status at the time of the extractions were not ascertained.

Generally, as supported by several studies,^26^,^29^,^30^ fewer extractions would be expected among the insured, a reflection of the better option of saving the tooth by endodontic treatment, as it would be at little or no cost to them. Although costs associated with RCTs vary across dental practices, they are high in comparison to tooth extraction as well as other minor dental procedures. Patients have been reported to stress the high costs of RCT with requests for it to be included among dental treatments covered by health insurance.^31^

One major finding in the present study was that a significant proportion (80%) of the subjects seen for RCT had dental health insurance. This finding supports the reason of the availability of health insurance as being responsible for the increase in the number of RCTs carried out in the center, as suggested by Idon and Yusuf (2018).^24^ This shows that dental insurance was a motivating factor for choosing to do RCT in our study. Several studies have associated possession of dental insurance to increased utilization of dental services.^7^,^10^,^15^,^27^ Others have also reported contrasting findings where dental insurance did not affect use of dental services. 25,32 Dental awareness may however, also be one of the reasons for this finding in our own study as majority (87%) of these subjects on dental insurance stated that they would still have chosen to do RCT in the absence of insurance. Janczarek et al. (2014) reported a lower proportion of 52% of subjects that would be willing to attempt the procedure despite the costs.^31^

Almost all (95.3%) the insured subjects were covered by the government funded health insurance scheme (NHIS) in this study. The number of subjects on employer-based dental insurance was minimal, while none was on private funded insurance. The explanation for this could be that the burden on the individual on the NHIS is negligible, but coverage available to individual subscribers as private insurance is expensive and thus uncommon in this environment. Also, depending on what works best and the stage of development, health care insurance systems seem to differ among countries.^22^ Obeidat et al.^6^ reported that only about half of Jordanian adults with dental insurance were government funded while 7.9% were on private dental insurance, and the rest (44.8%) were provided by insurance for those in the military and the university system.

Our study showed that males, subjects in the 35 –54-year age group, the widowed and subjects in the class IV occupation category presenting for RCT were more likely to be insured. None of these demographic characteristics were however found to significantly affect the subjects' insurance status and their presentation for RCT. A higher proportion (84.4%) of the subjects in the 35 – 54-year age range were insured. This age range would usually be made of adults in active public service and thus most likely to be insured in the NHIS program. Subjects in the older age group on the other hand would include a high proportion of retired adults who would no longer be on the government funded insurance (NHIS) program. Bhatti et al. (2007) reported a marked drop in insurance coverage among those aged above 65 years in Canada, and suggested to be due to stoppage of coverage by employers following retirement.^3^ Our study and the latter example show that the expensive nature of health insurance coverage for individual subscribers could explain why those in the retired age group would have lower proportion of insured.

The class of occupation as an indirect measure of socioeconomic status or income also did not significantly affect health insurance status and attendance for RCT. Although insignificant, the class IV occupation group, which is a low-income group, had a higher proportion of insured subjects. The Nigerian health insurance system includes coverage for both medical and dental services and is independent of the individual's income. This may also explain why none of the sociodemographic characteristics were found to have significantly affected the insurance status of subjects that were seen for RCT in this study. Although not directly related to endodontic treatment, Bhatti et al. (2007) reported that the amount of care received by Canadians increased with income.^3^ This is probably because Canadians are responsible for providing finance for their own dental care through private insurance or out-of-pocket payment. Higher income therefore correlates to dental insurance and more access to comprehensive dental care like endodontics. Similar reports have been seen in countries like Australia where dental insurance is majorly purchased privately, and being expensive, is dependent on socioeconomic status.^14^ This would explain why household income affected amount of specialist dental care received in these two studies. In contrast, Al-shammari et al. (2007) reported that household income was not considered by the subjects in their study to be a barrier to accessing the range of available dental services as they were provided free for Kuwaiti citizens.^33^ Here, dental insurance would not be necessary for assessing dental care of any type, and socioeconomic status would not also pose a barrier to any restorative dental treatment.

This study is limited by its cross-sectional design in the ability to report the observed associations between the subject variables and possession of dental insurance for RCT. Furthermore, the study did not assess direct relationship between household income and dental insurance status. This study however indicates the need for a more comprehensive study that would include samples from different dental centers in regions around the country with larger sample size, to allow for results that will reflect the effect of dental insurance on access to endodontic treatment and to dental treatments generally. With majority of the subjects in this study coming from the formal sector, it can also be suggested that the NHIS be sustained and the scope widened to cover individuals in the informal sector.

## Conclusion

Access to RCT was significantly affected by dental insurance. Cost of RCT is an important factor in the choice of treatment between RCT and extraction. The findings showed that occupation as a measure of socioeconomic status did not affect dental insurance status and presentation for RCT. Dental insurance had a greater effect on visiting for RCT among the low socioeconomic group, occupation class IV. Further studies with samples that are more representative of the population are required to assess the generalizability of this finding.
